# Development necessitates evolutionarily conserved factors

**DOI:** 10.1038/s41598-025-92541-4

**Published:** 2025-03-22

**Authors:** Paco C. K. Chow, Peter J. Bentley

**Affiliations:** Department of Computer Science, University College London, WC1E 6BT London, UK

**Keywords:** Computational science, Computational models, Machine learning, Computational biology and bioinformatics, Developmental biology, Evolution

## Abstract

Early-stage generalised transcription factors in biological development are often evolutionarily conserved across species. Here, we find for the first time that similar factors functionally emerge in an alternative medium of development. Through comprehensively analysing a Neural Cellular Automata (NCA) model of morphogenesis, we find multiple properties of the hidden units that are functionally analogous to early factors in biological development. We test the generalisation abilities of our model through transfer learning of other morphologies and find that developmental strategies learnt by the model are reused to grow new body forms by conserving its early generalised factors. Our paper therefore provides evidence that nature did not become locked into one arbitrary method of developing multicellular organisms: the use of early generalised factors as fundamental control mechanisms and the resulting necessity for evolutionary conservation of those factors may be fundamental to development, regardless of the details of how development is implemented.

## Introduction

The causes of evolutionarily conserved factors in biological development are likely to be complex. Evolution conserves those genes and proteins that are essential in the developmental processes of multicellular life, as even minor changes may produce nonviable results^1^. This is evident, for example, for the homeodomain factors, which play a fundamental role in biological development, throughout all known multicellular life^2,3^. These transcription factors (TFs) regulate large numbers of other genes in a temporal and spatially specific manner to regulate diverse developmental pathways^4,5^, including body plan specification and axis patterning^6^, cell fate determination and cell differentiation^7,8^, and embryonic stem cell maintenance^9^. Modifications to these factors can result in major changes, and thus we see evolutionary conservation across all eukaryotes, with indications that an ancient conserved functional domain may be shared across angiosperm and metazoan^10^. Evolutionary conservation is, by definition, the preservation of features by natural selection. But the constraints associated with development impose the need for such conservation. If development did not make use of generalised early TFs that are sensitive to modification, then evolutionary conservation might not be required at all. But given billions of years of experimentation, could evolution find no better solution? Does development necessitate evolutionarily conserved factors?

If false, we should view the role of evolutionarily conserved factors such as the homeodomain as merely an accident of one arbitrary method for generating multicellular organisms, that bears no more relevance than vestigial traits or spandrels visible in phenotypes, or the abundance of human endogenous retroviruses (HERVs) in the genome^11^. Perhaps the notion of such generalised early factors might be readily replaced with alternative or simpler methods of cellular control that might be more robust against disruption. But if true, then generalised early factors are another example of evolutionary optimization: a clever strategy devised early in the formation of life and fine-tuned to become an efficient control mechanism for all multicellular life on Earth. Perhaps as argued by Kauffman this clever method, which led to the formation of *Hox* and other early factors, created such “adjacent possibilities” that it helped cause the Cambrian explosion^12,13^. Not only would such factors be conserved in countless living examples as has been observed, but an entirely novel optimised process for multicellular development would - out of necessity - have to use a similar method resembling generalised early factors to function effectively, and these factors would have to be conserved in subsequent evolution.

In this work, we provide evidence to suggest that the latter may be the case. We use a model of development based on Neural Cellular Automata^14^ (NCA), which while similar in abstract to gene regulatory networks (GRNs), does not explicitly model genes. Instead, NCA models make use of a neural network to provide both inhibitory and excitory factors used to control artificial cellular development. Through interrogating the hidden representations of the NCA, we show for the first time that even in such a different model of development, we observe the emergence of early generalised factors (with some resemblance to biological homeodomain factors) that are evolutionarily conserved. This finding has profound implications. If early generalised factors are not merely an arbitrary way, but the only viable way to achieve multicellular development, we can then examine examples such as homeodomain factors in biology in a new light: while evolution may exploit them to enable morphological diversity^15^, the evolutionary conservation of many fundamental sequences is an inevitable consequence of development.

## Background

In biology, evolutionarily conserved early generalised factors like homeobox^4,5^, GATA^16^, and FOX proteins^17^ are transcription factors that can play important roles in development. A key defining property of evolutionarily conserved factors is the strong conservation of their function throughout evolution, from animals^18–20^ and plants^21,22^ to fungi^23–25^ and *Dictyostelium*^26,27^. Beyond this, evolutionarily conserved factors may share other functional properties. Firstly, some are active from the earliest stages of development. They are involved in early embryogenesis^28–30^ and play important roles in the development of tissues like the nervous system^31–33^, the cardiac system^34^, as well as in other developmental processes^30,35,36^. Secondly, their expression profiles show clearly defined spatial domains in different regions of the developing embryo, thereby playing a crucial role in the spatial patterning of tissue development^34,35,37–43^. Finally, loss-of-function results in major disruptions to morphological development. Notably, mutations to a subset of homeobox genes, known as *Hox* genes, can result in homeosis - the development of body structures in the wrong places^44,45^. Mutations in other homeobox genes can result in head involutions^46^, eye reductions^46–48^, limb malformations^40^, tooth agenesis^36^, disrupted development of tissues like the visceral mesoderm and heart^49,50^, and can also cause various genetic disorders and cancer^43,51–54^.

Despite their importance in biological development, there has been a lack of computational models that show their importance with regards to fundamental principles of development. Computational models of development attempt to model the process of biological development to a range of abstraction levels^55^. Models that are more biologically realistic tend to focus on specific developmental questions in isolation and require building in a large amount of biological knowledge. Examples of such models include gene circuit models^56^, reaction-diffusion models^57^, and multi-scale models like LBIBCell^58^, *EmbryoMaker*^59^, and MecaGen^60^. More abstract models of development like compositional pattern-producing networks^61^ and L-systems^62^ are able to model the development of large scale structures, but are not tied to the constraints of biology and so unable to model the functional importance of early generalised factors. The details of these models are discussed in the Supplementary Materials.

Studying development in a top-down approach, where a developmental behavior is specified and the system searches through the rules of cellular interactions that produce this behavior, have largely relied on evolutionary algorithms (EAs) with cellular automata (CA) to grow patterns. For example, Miller^63^ used Cartesian Genetic Programming, a type of EA, to grow a French flag pattern on a cellular automaton grid. Many other models have used other types of EAs to generate simple patterns and shapes of this sort^64–68^. However, this method only works for simple patterns with limited colours, and only small patterns can be generated. As shown by Elmenreich and Fehérvári^67^, models trained with CA-based EAs struggle to reproduce more complex images like the Mona Lisa - while the broad shape and colour scheme can be matched, the important intricate details are unable to be reproduced. Other studies have used one-dimensional CA to study more specifically sets of interaction rules that produce periodic morphogenetic patterns similar to that in fruitflies^69^. In general, using CA as models of development have been largely limited by the simplicity of the model.

With the advent of deep learning and neural networks, Neural Cellular Automata (NCA) models have developed to generate more complex patterns and model morphogenesis^14^. In this approach, cells are described by continuous variables and the update rule is parameterised by an artificial neural network. This means that instead of relying solely on a set of rules designed by hand, NCAs utilise trainable neural networks to update the state of each cell based on its neighbourhood. This integration allows the system to learn, adapt, and exhibit behaviors far more complex and versatile than classical CAs. Using gradient descent-based techniques, NCAs can be trained to achieve desired outputs or behavior based on input data. Mordvintsev et al.^14^ trained an NCA model to produce patterns of growth that stably converge to desired large-scale 2D morphologies. Starting from a single cell, the model is used to update the states of every cell on the grid for a number of iterations. The outcome of this is then compared to a target image of a gecko and a per-pixel loss is calculated. Backpropagation through time is used to update the parameters of the model until the model learns to grow the target image, maintain it without deformation, and be able to spontaneously perform self-repair if the image becomes damaged.

In effect, each cell models a biological cell, and the update rule models how the genome guides cell communication and growth. Despite the obvious simplicity in that the final pattern only exists on a 2D grid, the problem of guiding individual cell behavior based on the cell’s neighborhood’s states to form a globally coherent morphological form is in essence the same as the problem biological cells need to solve to develop into a multicellular organism in the 3D world. NCAs therefore explicitly model the development of entire anatomical structures but also contain internal representations that are amenable to examination and perturbation.

There have been many other follow up papers to the original Growing NCA model that explore this model further and extend this to modelling different aspects of morphogenesis. These are described in the Supplementary Materials. Despite the progress in this space, many models lack biological motivation and tend to ignore important biological properties of development. For instance, during embryonic development in invertebrate embryos, many maternal effect genes expressed in the mother’s ovaries produce maternal factors that act as morphogens - these are proteins specifically localised in different regions of the egg to set up pre-patterns and thereby initiate specific gene expression patterns from the zygotic genome^70^. However, all NCA models that have been developed simulate growing embryo in a vacuum and ignore the importance of the external environment which the embryo develops in, meaning that these models can only simulate the development of organisms in one orientation.

## Methods

### NCA model

**Fig. 1:**
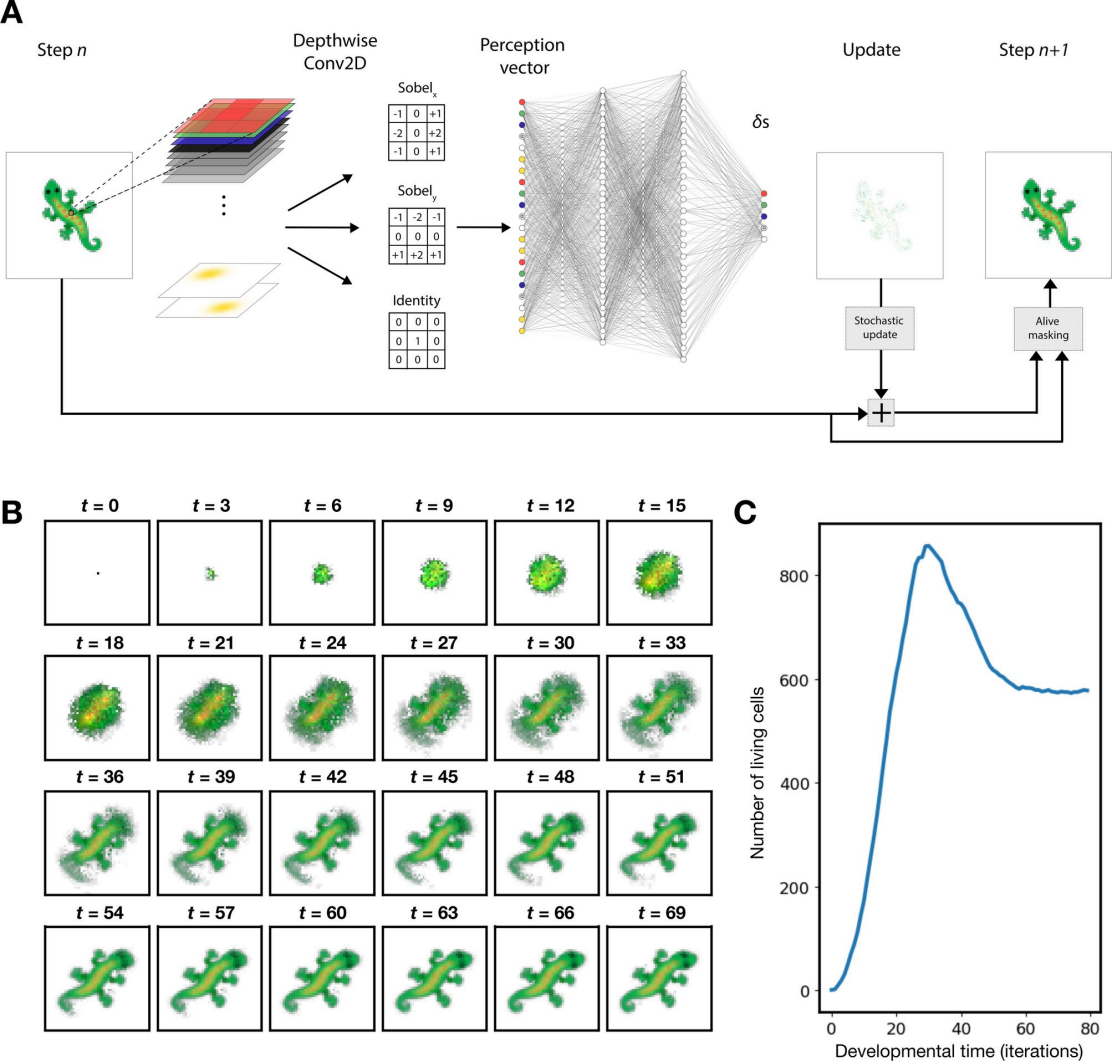
(**A**) NCA model architecture. Every cell consists of 16 model channels, the first 4 of which are the visible RGBA channels and the rest are hidden channels, as well as 2 environment channels. For every cell, a perception vector is created through depthwise convolution of fixed 3 × 3 filters. Each perception vector therefore contains information about the states of the cell and its neighbours. The perception vector is fed as input to a fully connected neural network with 2 hidden layers of 100 and 200 hidden units respectively, followed by a ReLU activation function. The output layer has 16 units and represents the updated states of that cell. This process is repeated in a recurrent manner until the end of the growth period. (**B**) Result after training with modulation. Growing a gecko with environmental modulation. (**C**) Number of living cells at every iteration of the developmental period.

Our NCA model is an extension of the Growing NCA model developed by Mordvintsev et al.^14^ As mentioned, one major limitation of the original model is that it is unable to grow rotation-invariant patterns (i.e. patterns that can be grown in different orientations). Our model simulates the maternal environment and achieves rotation-invariance growth by using the environment to guide the orientation with which the pattern develops, an approach more biologically plausible than alternative extensions of the model^71,72^. The architecture of the model is shown in Fig. 1A and is described in further detail in the Supplementary Materials.

In our model, cells exist on a regular Cartesian grid. The state of each cell is represented by a vector of 16 real values. Each value represents a channel, where the first 4 channels are visible RGBA channels, and the other 12 are hidden channels which the model is able to freely make use of to learn the task. At iteration 0, a seed cell is placed in the centre of the grid, which has all channels except RGB set to 1, and the rest of the grid is initialised with zeros. Cells then iteratively update their states using information collected from their 3 × 3 immediate neighborhood.

To implement an environment that the model can perceive, we introduced 2 extra channels to the grid. The state of the grid is therefore characterised by 16 ‘model channels’ that are part of the model, i.e. the model is able to perceive and output values to, as well as 2 ‘environment channels’ where the model is able to perceive values, but cannot output values to them. The 2 environment channels are used to set up an inherent polarity. One of which has a circular gradient in the anterior end and the other in the posterior. These simulate the two organising centres of maternal factors for example in fruitflies that establish the embryonic axes^73^. The model is able to perceive values in the environment channels, but are only able to output values for its own model channels, meaning it is unable to affect the environment. The model is trained to respect the polarity in the environment, developing into a pattern that has a head at the anterior gradient and a tail at the posterior one.

At every iteration of developmental time, the model undergoes a perception step, where each cell collects information about the state of its neighborhood using a 3×3 convolution with a fixed kernel, forming a perception vector. Cells update their states using a learned rule that is represented by a three-layer convolutional neural network. The neural network takes in the perception vector as input, transforms this vector through its hidden representations via units in hidden layers 1 and 2, and outputs the new states for the next iteration. Weights between units in different layers can be positive or negative, so every unit is capable of activating or inhibiting units in the next layer. Cell updates occur stochastically, thereby preventing the synchronisation of all cells updating simultaneously at every discrete time step, as this is not biologically realistic.

For the majority of our results (unless otherwise stated), we will be using a target image depicting a gecko (shown in Fig. 4A), which was similarly used in the original model^14^. (While alternative morphologies resembling plants or fungi could be used – perhaps resulting in better analogies to nature – for purposes of replicability we follow the original model.) Similar to the original paper^14^, we use a sample pool method to train our NCA such that the target pattern is set as an attractor. This means that our model is trained to grow the target pattern starting from a single seed cell, and then be able to stably maintain the target pattern, even after the target pattern is corrupted. Essentially, the sample pool method involves starting the training loop with a pool of seed states. Each seed state is a grid initialised with zeros except for a seed cell in the centre. We then use our model to grow a target pattern, and the outcome of this is updated in the pool. Thus, when training begins, the model is mainly tasked with growing the target pattern from a seed cell. As training progresses and more outcomes of training are returned to the pool, the model becomes tasked with growing the target pattern from the outcome of older training epochs. The result of this is that the model learns both to grow the pattern but also ensure that the pattern persists without deformation once it has been grown. Specific details and hyperparameters used for the training protocol is described in the Supplementary Materials.

## Results

### Model achieves rotation-invariant growth and exhibits biologically realistic developmental phases

After training with gradient descent, our model is able to utilise the orientation of the simulated maternal environment to guide the establishment of the anterior-posterior axis of the developing gecko. Examining the growth process in Fig. 1B, we find that our model shows distinct phases in the development period that interestingly seem to share visual similarities to specific phases of biological development: Expansion, differentiation, and pruning^74^. From iterations 0 to 9, the gecko forms a homogeneous-looking ball of cells, reminiscent to a blastula. The pattern then undergoes “differentiation” between iterations 9 and 30 as the homogeneous ball of cells differentiate into their respective structures. Finally, from iterations 30 to 60, the pattern is pruned as the number of cells decrease. Although the pruning phase is less visually apparent in Fig. 1B, it becomes clear when we plot the number of cells against developmental time (Fig. 1C). There is a clear peak at iteration 30, followed by a decline in the number of cells until the organism has fully developed into its final adult form at iteration 60. This behaviour of cellular overproduction followed by pruning is often observed in nature to sculpt precise body morphologies, for example during the formation of fingers in developing human embryos^75^. The period of growth between iterations 0 to 60 will be termed the ‘developmental period’ while iterations after are termed ‘adulthood’ (despite the visual similarities we recognize that the simplicity of this developmental system with its sequence of expansion, differentiation, and pruning, means that organogenesis might be a more apt analogy, rather than embryogenesis).

### Hidden units of model share similar properties with generalised early TFs

In biology, one method to understand the function of a protein is by visualising its activity in space and/or in time. By tagging proteins with fluorophores, biologists can image the localisation of a protein at a given snapshot in time or dynamically across a given time period and thereby infer a lot about its function^76,77^. Thus, to gain insights into how our model works, we perform an experiment similar to this by examining the pattern of activation of the hidden units of hidden layers 1 and 2 in the model across space and time.

**Fig. 2:**
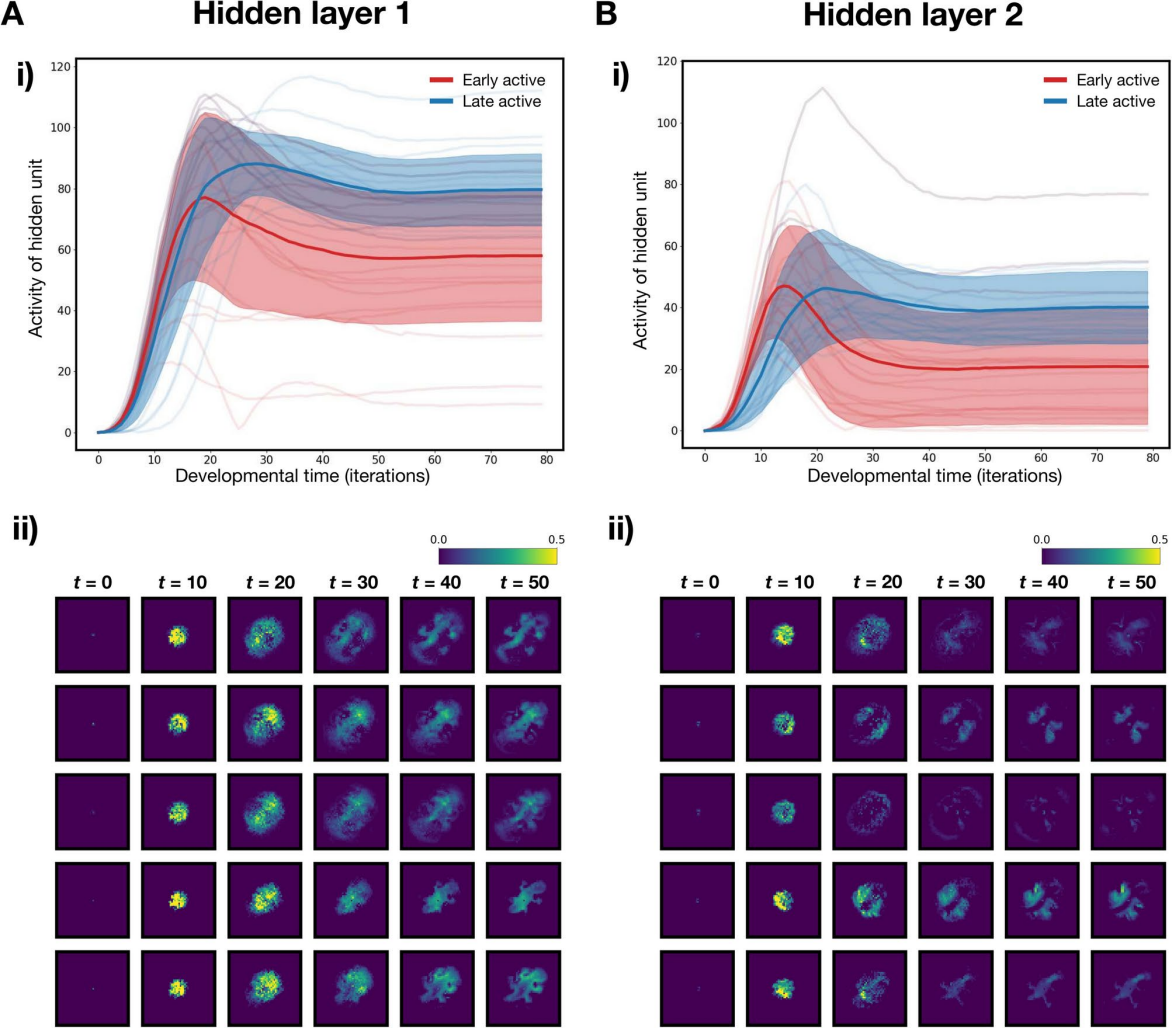
Temporal and spatiotemporal profiles of early active hidden units. Activity of hidden units is summed across every pixel on the grid at each iteration in time to give temporal activity profiles. (**A**)(i) Mean (thick line) and standard deviation (shaded) for the temporal activity profiles of 20 earliest active (blue) and late active (red) units are plotted. Temporal profiles of the 20 individual units for both categories are plotted in the background in their corresponding colours. ii) Spatial activity profiles of 5 earliest active hidden units in hidden layer 1 at different iterations of the developmental process. (**B**) Corresponding temporal (i) and spatial (ii) activity profiles in hidden layer 2.

We begin by visualising how the activity of the hidden units vary across time. For every iteration in time, we sum across a unit’s activation across all the pixels on the grid to obtain temporal activity profiles for each hidden unit. As mentioned, one interesting feature of our model is the growth of the ball of cells between iterations 0 to 9, as it is visually similar to cleavage in biological development. We can investigate this in greater detail by examining the most active hidden units up to iteration 9. We call such units ‘early active units’. The mean temporal activity profiles of the 20 earliest active units of hidden layer 1 and 2 are plotted in Fig. 2A(i) and B(i) respectively. These hidden units show strong activation peaks early in development, with varying levels of activity throughout the developmental period and into adulthood. These temporal profiles are reminiscent of the expression profiles of early-stage generalised transcription factors like homeobox genes. In contrast, the activity of late active units peak later in development (also shown in Fig. 2A(i) and B(i)), and are sustained at a higher level through adulthood.

To analyse these hidden units further, the activity profiles of the 5 earliest active hidden units in both hidden layers are visualised across space and time in the bottom panels of Figs. 2A(ii) and B(ii). Again, these hidden units show activity profiles that are similar to the expression profiles of early stage generalised factors, for example homeobox genes, patterning different regions of the developing embryo. In particular, early active units in layer 1 show stronger levels of maintained activation throughout adulthood, while early active units in layer 2 seem to only be transiently activated during early development.

**Fig. 3:**
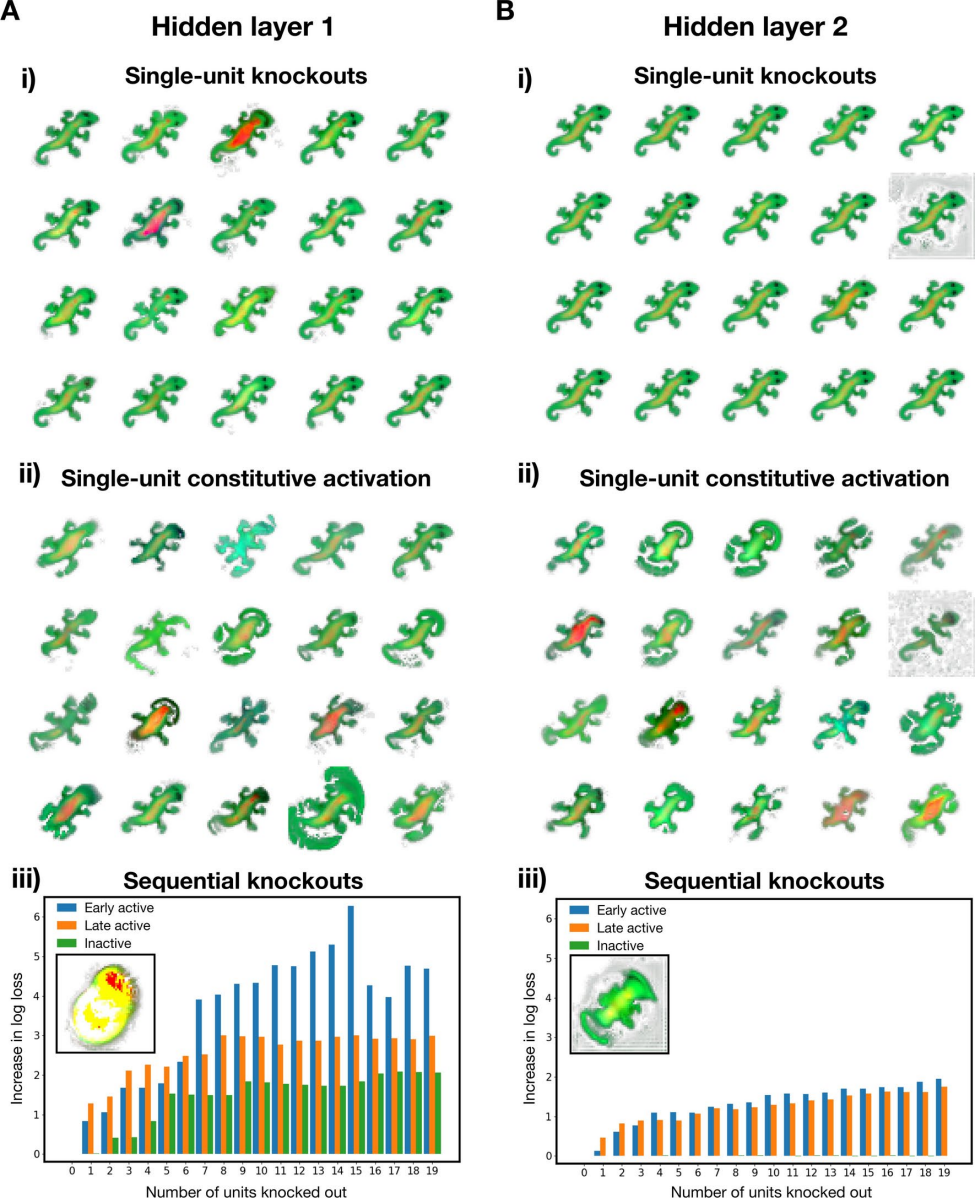
Perturbations show early active hidden units in hidden layer 1 are more important for morphological development. (**A**)(i) Outcome of development after single-unit silencing of earliest active hidden units in hidden layer 1. (ii) Outcome of development after single-unit constitutive activation of earliest active hidden units in hidden layer 1. Constitutive activation was performed by holding the activity of the hidden unit at 0.5 where cells are alive. (iii) Comparison of loss after sequentially knocking out earliest active hidden units (blue) compared to knocking out late active hidden units (orange) and inactive hidden units (green) in layer 1. Early active hidden units are defined as hidden units most active between iterations 0 to 9. Late active hidden units are hidden units most active between iterations 30 and 60 that are not early active units. Inactive hidden units are hidden units least active between iterations 0 to 9. Loss is defined as the sum of squared difference between developed pattern after 100 iterations of growth and reference gecko. Inset figure shows outcome of development after knocking out 20 earliest active hidden units in layer 1 is shown. (**B**)(i) Outcome of development after single-unit silencing of earliest active hidden units in hidden layer 2. (ii) Outcome of development after single-unit constitutive activation of earliest active hidden units in hidden layer 2. (iii) Comparison of loss after sequentially knocking out earliest active hidden units (blue) compared to knocking out late active hidden units (orange) and inactive hidden units (green) in layer 2. Inset figure shows outcome of development after knocking out 20 earliest active hidden units in layer 2.

One of the hallmarks of biological early generalised TFs such as homeobox is that knocking out genes results in disruption of anatomical structures, cancer, and various genetic disorders. To investigate whether these early active units show similar properties, we perform functional knock-out and constitutive activation experiments to perturb their normal activity throughout development. Here, hidden units are ‘knocked out’ by fixing their activity at zero for all pixels throughout the developmental period, and ‘constitutively activated’ by fixing their activity at 0.5 for all pixels throughout the developmental period. The result of individually knocking out the 20 earliest active units of hidden layers 1 and 2 are shown in Fig. 3A(i),B(i) respectively. From these grown geckos, it is clear that knocking out layer 1 early active units result in greater disruptions to anatomical structures than layer 2. While layer 2 perturbations show little changes in morphology compared to the ‘wild-type’, layer 1 perturbations show distortions of the head and eyes, disrupted patterns on the back and mild disruptions of body shape. Interestingly, we did not observe any homeosis phenotypes.

In contrast, constitutively activating each of the 20 early active units of layer 1 and 2 individually result in more drastic changes (Fig. 3A(ii),B(ii). The phenotypes show little differences between layer 1 and layer 2 perturbations. The head and tail are typically distorted, the patterns on the back are lost, and overgrowing of cells can be observed in some cases, reminiscent of a cancerous phenotype.

To quantify the effect of perturbations, we sequentially knock out increasingly more early active units and record the loss, comparing this with late active and inactive units as a control. We find that sequentially knocking out increasingly more early active units results in a much greater increase in loss compared to when late active units are knocked out (Fig. 3A(iii),B(iii)). The outcome of knocking out 20 early active units compared to knocking out late active and inactive units are also shown (inset Fig. 3A(iii),B(iii)). The phenotype in Fig. 3B(iii) shows little morphological structure while the phenotype in Fig. 3A(ii) retains broad structures like the head, body and tail. From these plots, it is clear that these early active units in hidden layer 1 play a more crucial role than other units in growing the target morphology, just as homeobox genes play a more crucial role than other genes in biological development. To summarise, early active units in hidden layer 1 are active from the earliest stages of development, show patterning of spatial domains during development, and results in the disruption of anatomical structures when perturbed, showing that they share many functional properties to early generalised TFs.

### Model performs effective transfer learning on new targets

**Fig. 4:**
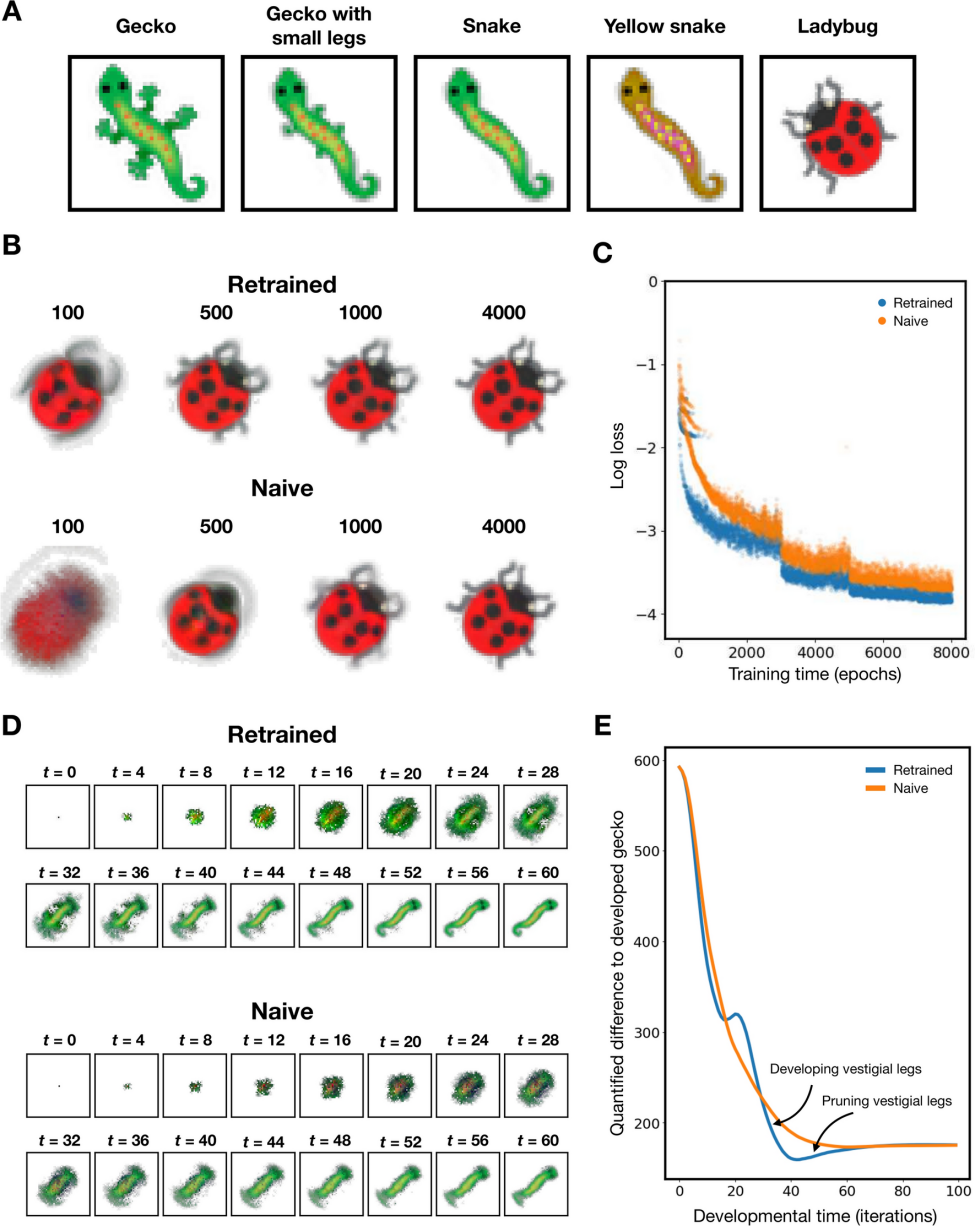
Generalisation to new targets. (**A**) Target patterns. From left to right: Gecko, gecko with small legs, snake, yellow snake, ladybug. (**B**) Outcome of development at different training epochs throughout retraining (top) and during naive model training (bottom). (**C**) Comparison of log loss throughout training between retrained model (blue) and naive model (orange). Loss is defined as the sum of squared difference between developed pattern and reference gecko. Retraining model on snake pattern results in appearance of vestigial leg-like structures during development. (**D**) Developmental process of retrained snake (top) and naively trained snake (bottom). (**E**) Quantifying the similarity between the development of a gecko and a snake. At every iteration of the developmental period, the developing snake using the retrained model (blue) and the naive model (orange) is compared to the developed pattern of a gecko by computing the sum of squared difference in visible channels between the developing snake and the developed gecko. This quantitatively reveals the appearance of vestigial legs in the retrained model represented as a dip in quantified difference between iterations 25 and 50 (indicated by arrows).

To test whether our model is able to generalise what it has learnt about growing a gecko to growing other morphologies, we apply transfer learning. This experiment essentially simulates how evolution adapts its solution from growing one body plan to a new one when its environment changes. New targets of varying anatomical similarity to the gecko are used for training. These range from a gecko with small legs, a snake, a yellow snake, to a ladybug (Fig. 4A). Using the model trained on the gecko, training is continued with the new target (we call this ‘retraining’).

Retraining our model results in successful and robust growth of all new target patterns (Fig. 4B, Supplementary Fig. 3). More importantly, retraining on the new targets results in significantly faster learning compared to naively training a model from scratch (Figs. 4B,C). Even with the ladybug target which is visually completely different to the original gecko, the model is able to grow much of the shape and patterns on the ladybug after just 100 iterations of retraining (Fig. 4B top panel). The log loss plots of retraining on a ladybug compared to naively training from scratch are compared in Fig. 4C. The difference between the two losses quantifies the amount of information training on a gecko transfers to growing a ladybug. This suggests that the model is able to adapt what it has learnt about growing a body into a new body plan, and implies that our model has learnt something fundamental about growing body plans.

In our model, given a fixed environment, the outcome of development is determined entirely by the parameters of the model. In other words, the different target patterns live in a high dimensional parameter space. During retraining, these parameters are modified into a new position in parameter space that encodes the new target. The trajectory of this movement in parameter space is governed by gradient descent, which is essentially the optimal way of modifying the parameters to reach the new target. The fact that retraining on a new morphological form results in much faster learning compared to naively training the model shows that the high dimensional parameter space is highly structured, and parameters that can grow coherent morphologies are clustered relatively close together. Thus, it requires less changes to the parameters to grow a new morphology when starting with parameters that are already able to grow a coherent pattern, compared to learning the appropriate parameters from randomly initialised ones.

Interestingly, when our model is retrained on the snake target, we find that during the developmental period, the model first grows leg-like structures from iteration 25 to 40, then prunes them off from iteration 40 to 50 (Fig. 4D top panel). In contrast, these vestigial legs are absent if the model is trained to grow a snake from scratch. To quantify this difference, we compute the sum of squared difference between the pixels of the RGBA visible channels for the developed gecko pattern and either the retrained snake or the naive snake at every iteration of development (Fig. 4E). Both models overlap during the first 20 iterations because the initial development phase is very similar, as well as after iteration 50 because both models develop into the same snake. However, the retrained model shows a dip between iterations 30 and 60, which corresponds to the appearance of vestigial legs as there is less difference with the gecko when these vestigial legs are present.

### Retrained model display conservation of early active units

In evolutionary developmental biology, the effects of evolution on development can be studied by examining the similarities in gene sequences and expression patterns for species with different evolutionary relationships^78^. This method has yielded many insights into the evolution of development, for example the finding that developmental strategies like the use of *Hox* genes to control the anterior-posterior patterning is conserved throughout evolution and is re-used in both fruitflies and mice^79^.

An analysis on the spatiotemporal activity profiles of hidden units is thus performed in the same way as before to gain more insights into how developmental strategies change following retraining. Here, we take the top 20 most active hidden units at every iteration during the developmental period of the original model trained on the gecko and compare this with the models retrained on different targets. We hypothesise that we should see a conservation of early active units, and that the more ‘evolutionarily conserved’ the retrained model is, the more similarity there should be with the original model in terms of the number of active hidden units conserved at every iteration.

**Fig. 5:**
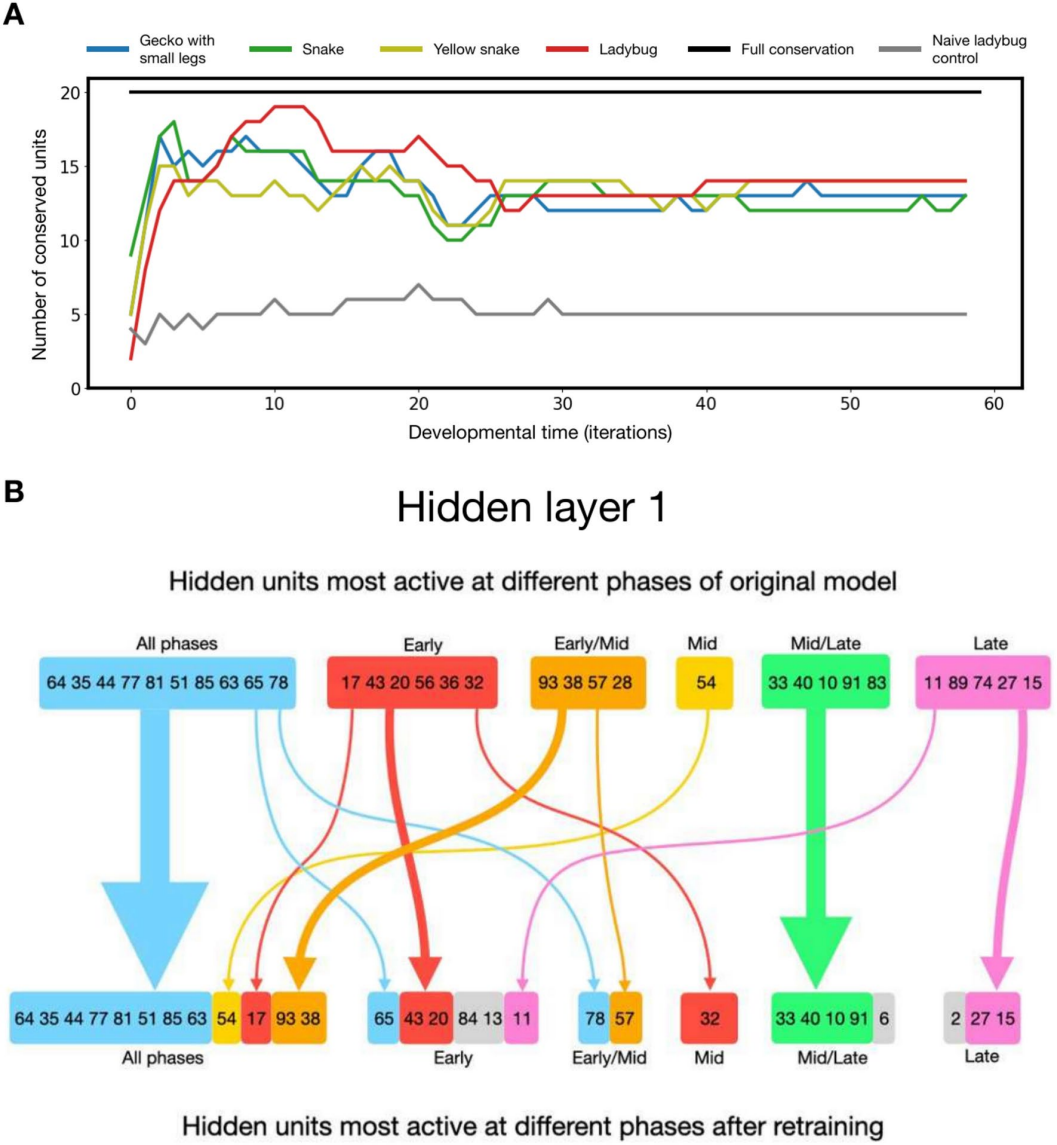
(**A**) Strong amount of conservation at each iteration across different targets. Conservation is measured by the number of similar hidden units that are most active at that developmental time between the original model and the retrained model. (**B**) Comparison of most active hidden units of layer 1 at every developmental phase for original model trained on the gecko pattern and after retraining on the ladybug pattern. Arrows show conservation of active hidden units after retraining. ‘Early’ denotes the developmental period of cell expansion between iterations 0 and 9. ‘Mid’ denotes the developmental period of differentiation between iterations 11 and 30. ‘Late’ denotes the developmental period of pruning between iterations 31 and 60.

Our results confirm the hypothesis (Fig. 5). We find that many of the active units are conserved after retraining on a new target. Interestingly, the amount of conservation did not correspond to how visually similar the new target is to the original gecko. Anatomically similar targets like the gecko with small legs, the snake, and the yellow snake, as well as anatomically distant targets like the ladybug all reuse around 13 units (Fig. 5).

To visualise the conservation of specific units, we compare the most active units at each developmental period for a given model, and compare the original model with the model after retraining on the ladybug target (Fig. 5B). As the ladybug is the most anatomically distant target from the gecko, this comparison will allow us to examine how the model is able to transfer its knowledge from the gecko to a new target that is visually completely different. From Fig. 5B, we can see that many early active units like units 64, 35, 44, 77, 81, 51, 85, 63 maintain high activity throughout the developmental process for both targets. Many other active units are conserved and used in the same development period, for example units 43 and 20 during early development, units 33, 40, 10, 91 during mid to late development, and units 27 and 15 during late development.

Another observation is that many units are conserved but are employed at different developmental periods after retraining. Interestingly, this phenomenon is also observed in *Hox* genes throughout evolutionary history, where a recurring theme in the field of evolutionary developmental biology is that highly conserved genes like *Hox* genes are reorganised and rewired for use in different developmental pathways to grow new body plans^80^, which often mean that *Hox* genes become employed in different developmental periods. For example, the *Hox* genes *ftz* and *zen* are expressed relatively later in development in chelicerates, myriapods, and crustaceans, but are expressed earlier for an early embryonic function in insects^81^. Meanwhile, variations in the expression timings of the *Hox* gene *Ubx* result in differences in leg length amongst different insects^82–84^.

A final observation is that active hidden units in layer 1 are strongly conserved at all developmental periods and are not limited to early active units. How, then, does the model grow a completely different morphology? The answer lies in hidden layer 2. When comparing the conservation of active hidden units in layer 2, we see that these units are more conserved during the early developmental phases compared to later phases (Supplementary Fig. 3). Out of the 20 hidden units most active in the early developmental stage of the original gecko model, 11 have been conserved after retraining on the ladybug target. On the other hand, out of the 20 hidden units most active in the late developmental stage of the original gecko model, only 1 is conserved. This is reminiscent of the comparisons between fins of fish and limbs of tetrapods, which show similar expressions of early-stage generalised TFs in the early budding phase, while different TFs are expressed in later stages^85^.

## Discussion

The process of biological development is fundamentally self-organizing and does not require external orchestration beyond the initial conditions provided by the organism’s environment and genetic material. The fundamental question of how a system composed of units that only interact locally is able to construct itself into a large-scale structure has long been solved by nature. Understanding how this process works is not only important for our understanding of biology, but will also accelerate advancements in regenerative medicine and synthetic bioengineering, as well as allow us to better understand how systems in general can robustly build up complexity from low-level components^86^.

Here, we have explored the relationship between development and the need for evolutionarily conserved, generalised early factors by considering a neural network trained via gradient descent to solve a 2-dimensional development problem. We have demonstrated that many of the functional properties of generalised early factors emerge in the hidden representations of this NCA. The hidden units of the neural network model exhibits functional characteristics of such factors, including: high activity early in development, spatial patterning, anatomical disruptions upon activity perturbation, and importantly, conservation following transfer learning on different morphologies. This is the first demonstration of the emergence of evolutionarily conserved factors in an alternative medium of development.

The early active units in our model show many interesting similarities and differences to early generalised factors in biology that are worth commenting on. Firstly, while we showed that knocking out early active units resulted in morphological defects similar to the results of knocking out homeobox genes, no homeotic phenotypes were observed. This may be due to the simplicity of our model and the fact that our model does not utilise metameric structures during development. By modifying architectural components of our model, it could be possible to achieve the invention of reusable modules and possibly homeosis upon perturbation, but this needs to be explored further in the future.

Secondly, we found that when the model has previously learnt how to grow a gecko and is now tasked with growing the same pattern but with removed structures (a snake), the model simply grows the gecko form it has already learnt and then prunes off the legs to form the snake. This recycling of anatomical modules to develop new body plans is precisely the strategy of biological evolution. In fact, limb loss or reduction are hallmarks of snake evolution. Primitive snakes like pythons and boas retain tiny hind leg bones buried in muscles towards their tail. These tiny legs are known as vestigial legs and reflect the fact that they have descended directly from lizards^87,88^. Thus, the fact that ‘evolution’ in our model and biological evolution both employ the recycling of anatomical modules to produce new body forms is another evidence to the fact that the strategy our model employs to grow a body is fundamentally similar to how biological development works. This is not a trivial finding and shows that the parameter space by which gradient descent operates to find parameters that can generate a new target pattern has deep similarities to the parameter space biological evolution operates on to generate new body plans.

In our model, we also find greater conservation in early developmental stages compared to later ones. This suggests that just like in biology, growing patterns requires similar early developmental strategies of cell proliferation and establishment of the major embryonic axes, while the specific pattern that is grown depends on later developmental strategies like differentiation into appropriate body structures and pruning of unnecessary structures, which will be different depending on how different the target pattern is. As this is similar to the case in natural evolution^85^, one explanation for why modifying parameters of our model to grow a new target through retraining can result in developmental features seen in evolution is that both our model and evolution operate on similar developmental strategies. To adapt a body plan to grow a snake instead of a gecko, for example, the easiest strategy is to make little modifications to the early developmental stages and only prune off the legs after they are grown.

We emphasise that we are making no claims about the evolutionary history of these biological organisms. Indeed, our decision to train on a gecko pattern before retraining on other patterns like a ladybug are purely arbitrary, and we would expect the same results to hold for training and retraining on any different types of pattern as there is nothing inherently special about the gecko pattern. The example of the loss of limbs in snakes was also chosen specifically to show that there can be visual similarities between biological evolution and our simulation of evolution in the sense that they can both show similar vestigial structures. Similar results are likely given retraining on any target pattern that has parts removed from the original trained pattern.

Finally, it should be noted that biological evolution operates in a fundamentally different way to how we simulate evolution here. Biological evolution has no end-goal - morphological changes arise due to random genetic events that produce neutral or adaptive consequences within an ecological context. In contrast, our training process directs the system towards a target final form, meaning that the “fitness measure” is fixed and predetermined unlike the moving target of natural selection. Despite this, biological evolution and ‘evolution’ in our system is similar in the sense that the system needs to develop a new morphology. Our key finding is that the reuse of early active units to grow very different morphologies shows that the model is adapting developmental strategies to grow new body forms, resembling the way that biological evolution reuses developmental strategies such as homeodomain proteins to grow different body forms. The ability to generalise to growing different target patterns was not part of the training process, so it is not obvious at all that when retraining our model on a different target pattern, that many of the same early active units would be conserved. The fact that in both biological development and our alternative model of development, the use of evolutionarily conserved factors that have similar functional properties suggests to us that perhaps there is an optimal strategy to development that both nature and our model have discovered. In 1992, Tierra demonstrated the emergence of parasites and predators in a computational model of evolution^89^. In 1994, Evolved Virtual Creatures demonstrated that an evolutionary algorithm can result in the evolution of natural looking and behaving forms^90^. In a similar vein, our work suggests that evolutionarily conserved factors may be fundamental to development, regardless of the implementation details of the developmental process.

## Supplementary Information

Supplementary Information.

## Data Availability

The datasets generated and analysed during the current study are openly available in the Neural-CA repository at https://github.com/pacochow/Neural-CA.
