# Supplementary material for: Development necessitates evolutionarily conserved factors
Source: Sci Rep. 2025 Mar 22;15:9910. doi: 10.1038/s41598-025-92541-4 (PMC11929755; doi:10.1038/s41598-025-92541-4)
Supplement: Supplementary file 1 — Supplementary Information. [file 41598_2025_92541_MOESM1_ESM.pdf]

Supplementary Materials

1 Supplementary background

1.1 Computational models of development

Existing computational models of development have attempted to model the process of biological development to a range of abstraction levels [1] yet have been unable to show the importance of early-stage transcription factors with regards to fundamental principles of development. Biologically realistic models are typically used to model specific developmental processes and require hard-coding biological details into the model. For example, gene circuit models were used to investigate the regulation circuitry between FGF, retinoic acid, and the homeobox genes *Meis1/2*, *Hoxa11* and *Hoxa13* in the development of the mouse limb bud [2]. By using a data-driven approach to infer the genetic circuit most likely to reproduce wild-type expression patterns of the transcription factors, the authors were able to show how homeobox patterning of the developing limb bud can be achieved by known gradients of upstream factors. Similarly, [3] used a stochastic simulation algorithm to model the importance of interactions between *Hox* genes in the development of rhombomeres, a key event that occurs during vertebrate hindbrain development. Other models used in developmental biology include reaction-diffusion models, which also require tailoring the parameters of the model to a very specific biological process. A good example of this is by [4] who simulated a reaction-diffusion system on a 2D mesh shaped as a developing mouse handplate and showed the mechanism by which the patterning of *Hox* genes can regulate digit patterning.

More recently, improvements in computing power have allowed for the development of multi-scale models like LBIBCell [5], *EmbryoMaker* [6], and MecaGen [7] that are able to model the dynamics of the cell's biomechanical behaviors coupled to the intracellular gene expression dynamics through an explicit gene regulatory network, making them useful for testing hypotheses regarding morphogenetic events during embryonic development. While these models are able to model a range of developmental processes to a high level of detail including epithelial differentiation, epiboly, gastrulation, these models are not situated at the appropriate abstraction level to explore the importance of homeodomain factors with regards to the development of entire anatomical structures.

Other models of development include the Cellular Potts model [8, 9] and Fleischer and Barr's simulation testbed of multicellular development [10]. In these models, multiple developmental mechanisms are implemented to enable different forms of interactions between discrete cells. While these models are able to simulate a range of multicellular behaviors including cell migration, cell differentiation, gradient following, and lateral inhibition, these models are only able to study development from a bottom-up approach – how changing the rules of interaction amongst cells results in the emergence of multicellular behavior. The difficulty of designing rules and functions that will result in the growth of a specific target pattern means that it is extremely difficult to study how the properties of the artificial genes or cell state parameters contribute to the development of different patterns.

More abstract models of development are able to model holistic aspects of development, such as the development of large scale structures. However, as they are not constrained by biological details, they can be less informative in understanding how development is coordinated by the biology. For example, compositional pattern-producing networks (CPPNs) have been used to evolve soft robots with diverse, interesting morphologies that can exhibit complex locomotion

behaviors [11], yet the model abstracts away the process of growth, meaning that there is no explicit simulation of development over time. Other abstract models like L-systems [12] model multicellular organisms as an array of symbols where each symbol is iteratively substituted according to a set of algorithmic rules. These substitutions represent cell divisions, cell death, or changes in cellular states, and depends on inputs from neighbouring cells or the cell state itself. This means that L-systems are able to test relationships between recursive local rules of development and the global properties of the developed system, and has largely been used to model the development of the filamentous bacterium *Anabaena catenula* and different aspects of plant development [13]. However, the recursive nature of the local rules that are implemented in L-systems mean that they are limited to modelling filamentous and branching structures, and are less able to test the role of specific genes in general principles of development.

1.2 NCA models

Follow up papers to the original Growing NCA model [14] have explored and extended the model to different aspects of morphogenesis. [15] and [16] extended the model to grow 3D morphologies and soft robots respectively. [17] generalised these results from NCAs to graph CAs. [18] and [19] explored the manifold of the NCA parameter space with autoencoders. [20] and [21] developed methods of adversarial attacks to control the self-organisation behavior of the model. [22] incorporated an information-theoretic metric into the loss function to improve cell coordination for achieving a collective task. [23] extended the model to grow neural networks that can solve common reinforcement learning tasks. [24] used encoded goal vectors to allow the model to continuously direct the growth processes of the cells, allowing the NCA to grow dynamic morphologies. [25] and [26] trained NCA to learn dynamic spatio-temporal patterns and dynamic textures on 3D meshes respectively. [27] trained the model with internal and external signals that triggers the model to develop into different morphologies. [28] used two interacting NCAs that operate on separate scales to model the emergence of patterns across scales. Notably, one recent model by [29] aimed to incorporate more biological details by explicitly using gene regulatory networks in the architecture of their NCA, allowing them to examine how gene mutations and gene interactions affect development. However, they were only able to train it to grow very simple target patterns that do not resemble organisms.

2 Supplementary Methods

The algorithm of the model is presented in Algorithm S1.

Algorithm S1 NCA model

Input: State $s_t \in \mathbb{R}^{50\times 50\times C}$, where C is the number of input channels
$p_t \leftarrow$ Perceive state
$u_t \leftarrow 50 \times 50$ random boolean array
$s_{t+1} = s_t + u_t * (relu(relu(p_t W_0 + b_0)W_1 + b_1)W_2 + b_2)$
Set all channels of empty cells to zero
Output: State $s_{t+1} \in \mathbb{R}^{50\times 50\times 16}$

Grid

Cells exist on a regular Cartesian grid. The state of each cell is represented by a vector of 16 real values. Each value represents a channel, where the first four channels are visible RGBA channels, and the other 12 are hidden channels which the model is able to freely make use of to learn the task. At iteration 0, a seed cell is placed in the centre of the grid, which has all channels except RGB set to 1, and the rest of the grid is initialised with zeros. Cells then iteratively update their states using information collected from their 3×3 immediate neighborhood.

$$s = [s^0 = R, s^1 = G, s^2 = B, s^3 = A, ..., S^{C-1}]$$

where s is the cell state, C is the number of channels and the first four channels represent a visible RGBA image. The A channel determines whether a cell is currently alive or empty. Cells are considered alive if they have $A > 0.1$ or at least one alive cell in its 3×3 neighborhood. Other cells are considered empty.

To implement an environment that the model can perceive, we introduced 2 extra channels to the grid. The state of the grid is therefore characterised by 16 'model channels' that are part of the model, i.e. the model is able to perceive and output values to, as well as 2 'environment channels' where the model is able to perceive values, but cannot output values to.

The two environment channels are used to set up an inherent polarity. One of which has a circular gradient in the anterior end and the other in the posterior. These simulate the two organising centres of maternal factors in fruitflies that establish the embryonic axes [30]. The model is able to perceive values in the environment channels, but are only able to output values for its own model channels, meaning it is unable to affect the environment.

To ensure that only living cells are able to perceive these gradients, we modify the growth process such that at every time step, the values of the environment channels are multiplied by the corresponding values of the A channel from the previous time step, so that only living cells have access to processing the environment. The model is trained to respect the polarity in the environment, developing into a pattern that has a head at the anterior gradient and a tail at the posterior one (see below for details).

Perception

Before the update step, each cell collects information about the state of its neighborhood using a 3×3 depthwise convolution with a fixed kernel. Per-channel Sobel filters K_x, K_y are used as the kernel for the x- and y-axis respectively. The Sobel filters are a popular edge detection kernel [31] and act in this case to estimate the state gradients along their respective axis, resulting in a gradient vector for the x-axis and one for the y-axis for each cell. The angle of perception can be modified by setting θ to any value other than 0, but for our purposes θ is kept at 0. These vectors are concatenated with the cell state vector to form a perception vector that is fed into the neural network for the update step. This perception vector carries all the information about the states of the cell and its neighbouring cells.

$$p_t = concat\Big(s, (K_x \cos\theta - K_y \sin\theta) * s, (K_x \sin\theta + K_y \cos\theta) * s\Big)$$

where s denotes the cell's state, θ denotes the angle of perception, and K_x and K_y are given by

$$K_x = \begin{bmatrix} -1 & 0 & 1 \\ -2 & 0 & 2 \\ -1 & 0 & 1 \end{bmatrix}$$

$$K_y = {K_x}^T$$

Update rule

Cells update their states using a learned rule that is represented by a three-layer convolutional neural network that uses 1×1-convolutions and ReLU nonlinearities. The neural network takes in the perception vector as input and outputs the new states for the next iteration. Using a convolutional neural network with 1×1-convolutions increases computational efficiency since every cell on the grid can be updated in parallel, as opposed to sequentially feeding in perception vectors into a fully connected neural network for every cell. The update rule is given by

$$s_{t+1} = s_t + relu(relu(p_t W_0 + b_0)W_1 + b_1)W_2 + b_2$$

Parameters W_0, b_0, W_1, b_1, W_2, b_2 have shapes (54, 100), (100), (100, 200), (200), (200, 16) and (16) respectively, which gives a total of 28916 parameters. W_0 and W_1 are initialised with uniform Xavier initialisation. b_0, b_1, W_2 and b_2 are initialised to zeros so that the model only outputs zeros in the beginning of training. The ReLU function is not used for the output of the final layer as the neural network outputs incremental updates to the cell states which means it must be able to output positive and negative numbers to add or subtract from the state.

Cell updates occur stochastically. At each NCA step, cells are updated with probability p_{upd} ($p_{upd} = 0.5$ is used for training). This stochasticity prevents the synchronisation of all cells updating simultaneously at every discrete time step, as this is not biologically realistic.

All channels of empty cells are explicitly set to 0 at every time step (alive masking) to prevent empty cells from performing any computations and carrying any hidden states.

Training

For the majority of our results (unless otherwise stated), we use a target image depicting a gecko, which was similarly used in the original model [14]. This target image is shown in Fig. 4A (main paper) and has dimensions 50×50 pixels. Just as in the original paper [14], we use a sample pool method to train our NCA such that the target pattern is set as an attractor. This means that our model is trained to grow the target pattern starting from a single seed cell, and then be able to stably maintain the target pattern, even after the target pattern is damaged (for example, if the tail is 'cut off' by setting the values of the channels in that area to 0). Essentially, the sample pool method involves starting the training loop with a pool of seed states. Each seed state is a grid initialised with zeros except for a seed cell in the centre. We then use our model to grow a target pattern, and the outcome of this is updated in the pool. Thus, when training begins, the model is mainly tasked with growing the target pattern from a seed cell. As training progresses and more outcomes of training are returned to the pool, the model becomes tasked with growing the target pattern from the outcome of older training epochs. The result of this is that the model learns both to grow the pattern but also ensure that the pattern persists without deformation once it has been grown. We describe how this sample pool is implemented in detail below for clarity.

Before training, we define a pool of 1024 seed states to start the iterations from. At the start of each training epoch, we sample a batch of 8 samples from this pool which we use in our training step, and replace the highest-loss sample in this batch with the original single-pixel seed state. This prevents catastrophic forgetting once all seed samples in the pool have been replaced, and ensures that the model is still able to grow the target pattern from the seed state. To encourage our model to be able to regenerate damaged patterns, we damage the three lowest-loss sample at the start of each training epoch by setting a random circular region within the pattern to zeros.

Before initiating the development stage, we uniformly sample 8 orientations between 0 and 2π (one orientation for every image in the batch) at the start of every training epoch. The pattern should be grown such that its anterior-posterior axis is aligned along that orientation, so we rotate the environment and the target patterns accordingly.

Then, we uniformly sample a random number of CA steps from the range [64, 96] and recursively run our model starting from the seed state for that number of time steps. At the last time step, we apply pixel-wise L2 loss between RGBA channels in the grid and the target pattern. Backpropagation through time is used to optimise this loss and learn the local update rule that satisfies the global objective of growing the target pattern. The Adam optimization algorithm is used to perform backpropagation with parameters learning rate $\alpha = 0.001$ and weight decay $\lambda = 0.0003$. At the end of the training epoch, we update the pool by replacing samples in the pool that were sampled for the batch with the output states from the training step over this batch. The model is trained for 8000 training epochs (the log loss plot from training this base model is shown in Supplementary Fig. S1). A multi-step learning rate scheduler is used to decay the learning rate by $\gamma = 0.3$ when the training epoch reaches milestones of 3000, 5000, and 7000. These hyperparameters are similar to those used in Mordvintsev et al. [14], and have been tuned slightly to give marginally better training results. Overall, hyperparameter tuning does not seem to significantly affect the training results of the model, so explorations of different hyperparameters have not been

documented.

The training loop is presented in Algorithm S2 below. Differences with the original Growing NCA algorithm [14] are indicated with a $\star$.

Algorithm S2 Model training loop

Initialise: Pool of 1024 seed states *pool*, target pattern y, base environment *base_env*, where $pool \in \mathbb{R}^{1024\times50\times50\times16}$, $y \in \mathbb{R}^{50\times50\times4}$, $base\_env \in \mathbb{R}^{\times50\times50\times2}$ $\star$
for epoch = 1 to 8000 **do**
    Sample 8 states from *pool*
    Identify the highest-loss sample in the batch
    Replace highest-loss sample with single-pixel seed state
    for each of the three lowest-loss samples **do**
        Set a random circular region within the sample to zeros
    for $i = 1, ..., 8$ **do**
        Sample $\gamma \in \mathbb{R}$ where $\gamma \sim \mathcal{U}(0, 2\pi)$ $\star$
        $y_i \leftarrow$ rotate y by γ $\star$
        $base\_env_i \leftarrow$ rotate base environment by γ $\star$
        $state_{i,0} \leftarrow$ seed state
        Sample $n \sim \mathcal{U}(64, 96)$
        for $t = 1, ..., n$ **do**
            $living\_cells \leftarrow state_{i,t}[3]$ $\star$ ▷ *α channel of state*
            $env_{i,t} \leftarrow living\_cells * base\_env_i$ $\star$ ▷ *Modulate environment*
            $full\_state_{i,t} \leftarrow \text{concat}[state_{i,t}, env_{i,t}]$ $\star$
            $state_{i,t} = \text{model}(full\_state_{i,t})$ $\star$
            if $t = n$ **then**
                $\hat{y}_i \leftarrow state_{i,n}[0:4]$ ▷ *RGBA channels of state*
                $loss_i = ||\hat{y}_i - y_i||_2^2$ $\star$
    Compute mean loss across batch
    Backpropagate through time
    Replace the batch samples in the pool with the outputs from the training step

3 Supplementary Figures

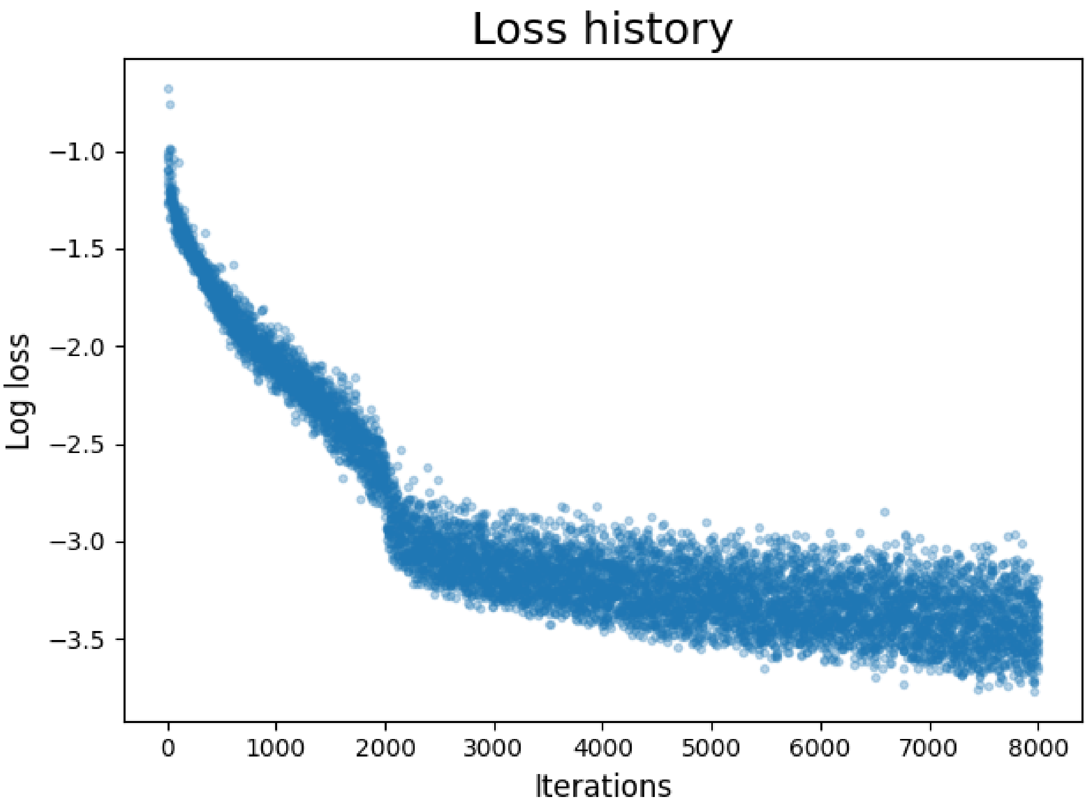

Figure S1: Log loss plot from training of original Growing NCA model.

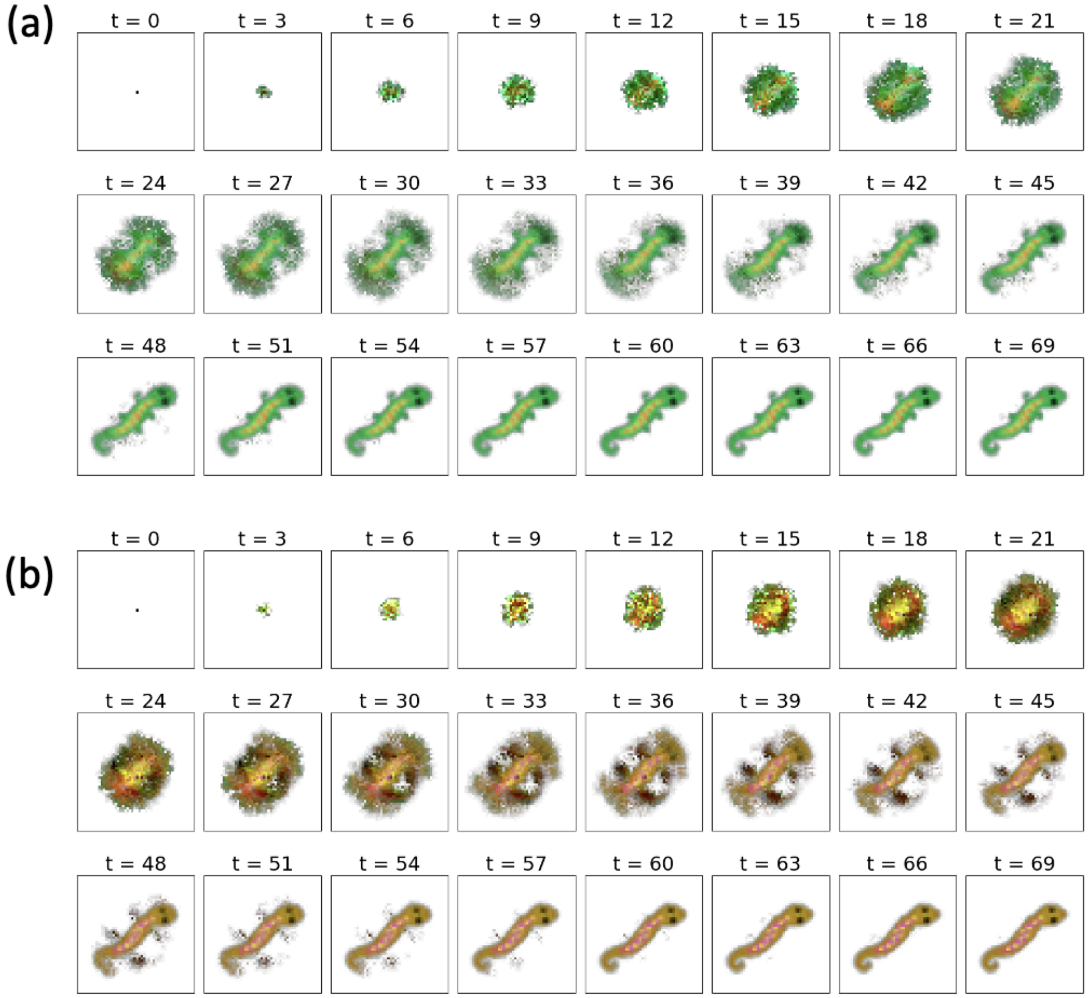

Figure S2: Developmental process after retraining on gecko with small legs (a) and yellow snake (b).

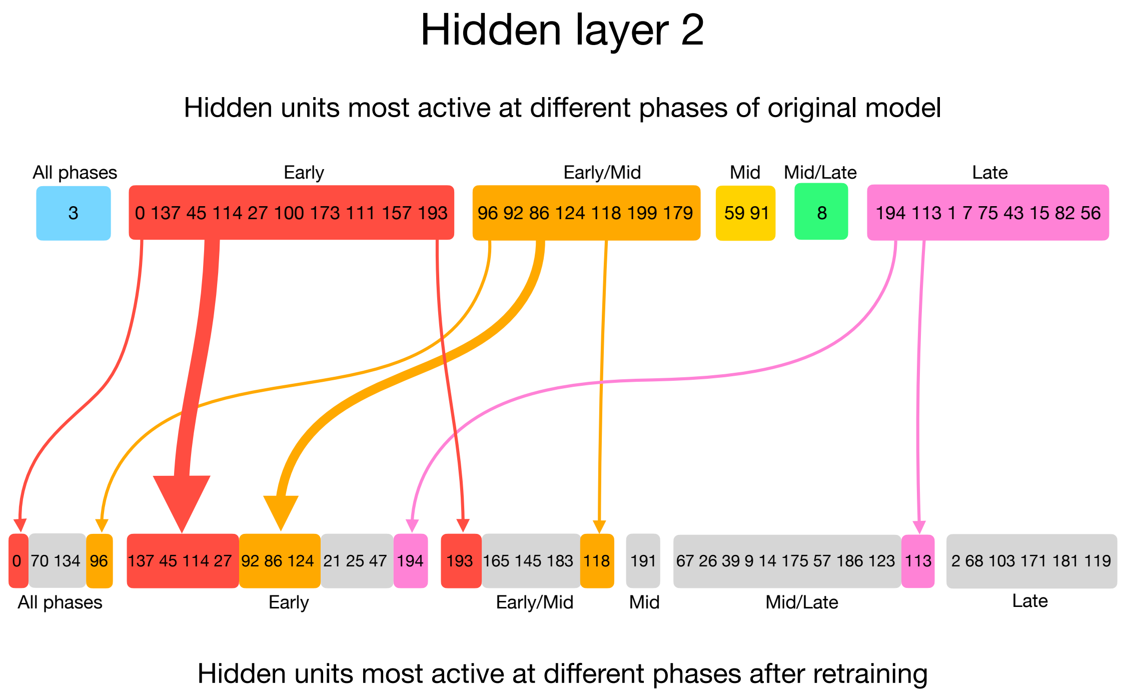

Figure S3: Comparison of most active hidden units of layer 2 at every developmental phase for original model trained on the gecko pattern and after retraining on the ladybug pattern. Arrows show conservation of active hidden units after retraining. 'Early' denotes the developmental period of cleavage between iterations 0 and 9. 'Mid' denotes the developmental period of differentiation between iterations 11 and 30. 'Late' denotes the developmental period of pruning between iterations 31 and 60.

References

[1] James Sharpe. Computer modeling in developmental biology: growing today, essential tomorrow. *Development*, 144(23):4214–4225, December 2017. ISSN 0950-1991. doi: 10.1242/dev.151274. URL `http://dx.doi.org/10.1242/dev.151274`.

[2] Manu Uzkudun, Luciano Marcon, and James Sharpe. Data-driven modelling of a gene regulatory network for cell fate decisions in the growing limb bud. *Molecular Systems Biology*, 11(7), July 2015. ISSN 1744-4292. doi: 10.15252/msb.20145882. URL `http://dx.doi.org/10.15252/msb.20145882`.

[3] Jason Kastner, Jerry Solomon, and Scott Fraser. Modeling a hox gene network in silico using a stochastic simulation algorithm. *Developmental Biology*, 246(1):122–131, June 2002. ISSN 0012-1606. doi: 10.1006/dbio.2002.0664. URL `http://dx.doi.org/10.1006/dbio.2002.0664`.

[4] Rushikesh Sheth, Luciano Marcon, M. Félix Bastida, Marisa Junco, Laura Quintana, Randall Dahn, Marie Kmita, James Sharpe, and Maria A. Ros. Hox genes regulate digit patterning by controlling the wavelength of a turing-type mechanism. *Science*, 338(6113):1476–1480, December 2012. ISSN 1095-9203. doi: 10.1126/science.1226804. URL `http://dx.doi.org/10.1126/science.1226804`.

[5] Simon Tanaka, David Sichau, and Dagmar Iber. Lbibcell: a cell-based simulation environment for morphogenetic problems. *Bioinformatics*, 31(14):2340–2347, March 2015. ISSN 1367-4803. doi: 10.1093/bioinformatics/btv147. URL `http://dx.doi.org/10.1093/bioinformatics/btv147`.

[6] Miquel Marin-Riera, Miguel Brun-Usan, Roland Zimm, Tommi Välikangas, and Isaac Salazar-Ciudad. Computational modeling of development by epithelia, mesenchyme and their interactions: a unified model. *Bioinformatics*, 32(2):219–225, September 2015. ISSN 1367-4803. doi: 10.1093/bioinformatics/btv527. URL `http://dx.doi.org/10.1093/bioinformatics/btv527`.

[7] Julien Delile, Matthieu Herrmann, Nadine Peyriéras, and René Doursat. A cell-based computational model of early embryogenesis coupling mechanical behaviour and gene regulation. *Nature Communications*, 8(1), January 2017. ISSN 2041-1723. doi: 10.1038/ncomms13929. URL `http://dx.doi.org/10.1038/ncomms13929`.

[8] François Graner and James A. Glazier. Simulation of biological cell sorting using a two-dimensional extended potts model. *Physical Review Letters*, 69(13):2013–2016, September 1992. doi: 10.1103/physrevlett.69.2013. URL `https://doi.org/10.1103/physrevlett.69.2013`.

[9] P. Hogeweg. Shapes in the shadow: Evolutionary dynamics of morphogenesis. *Artificial Life*, 6(1):85–101, January 2000. doi: 10.1162/106454600568339. URL `https://doi.org/10.1162/106454600568339`.

[10] Kurt Fleischer and Alan Barr. A simulation testbed for the study of multicellular development: The multiple mechanisms of morphogenesis. *Artificial Life III*, 02 1997.

[11] Nick Cheney, Robert MacCurdy, Jeff Clune, and Hod Lipson. Unshackling evolution. *ACM SIGEVOlution*, 7(1):11–23, August 2014. doi: 10.1145/2661735.2661737. URL `https://doi.org/10.1145/2661735.2661737`.

[12] Aristid Lindenmayer. Mathematical models for cellular interactions in development i. filaments with one-sided inputs. *Journal of Theoretical Biology*, 18(3):280–299, March 1968. doi: 10.1016/0022-5193(68)90079-9. URL `https://doi.org/10.1016/0022-5193(68)90079-9`.

[13] Przemyslaw Prusinkiewicz, Mikolaj Cieslak, Pascal Ferraro, and Jim Hanan. *Modeling Plant Development with L-Systems*, page 139–169. Springer International Publishing, 2018. ISBN 9783319990705. doi: 10.1007/978-3-319-99070-5_8. URL `http://dx.doi.org/10.1007/978-3-319-99070-5_8`.

[14] Alexander Mordvintsev, Ettore Randazzo, Eyvind Niklasson, and Michael Levin. Growing neural cellular automata. *Distill*, 2020. doi: 10.23915/distill.00023. https://distill.pub/2020/growing-ca.

[15] Shyam Sudhakaran, Djordje Grbic, Siyan Li, Adam Katona, Elias Najarro, Claire Glanois, and Sebastian Risi. Growing 3d artefacts and functional machines with neural cellular automata, 2021.

[16] Kazuya Horibe, Kathryn Walker, and Sebastian Risi. Regenerating soft robots through neural cellular automata, 2021.

[17] Daniele Grattarola, Lorenzo Livi, and Cesare Alippi. Learning graph cellular automata, 2021.

[18] Alejandro Hernandez, Armand Vilalta, and Francesc Moreno-Noguer. Neural cellular automata manifold. In *Proceedings of the IEEE/CVF Conference on Computer Vision and Pattern Recognition (CVPR)*, pages 10020–10028, June 2021.

[19] Rasmus Berg Palm, Miguel González Duque, Shyam Sudhakaran, and Sebastian Risi. Variational neural cellular automata. In *International Conference on Learning Representations*, 2022. URL `https://openreview.net/forum?id=7fFO4cMBx_9`.

[20] Ettore Randazzo, Alexander Mordvintsev, Eyvind Niklasson, and Michael Levin. Adversarial reprogramming of neural cellular automata. *Distill*, 2021. doi: 10.23915/distill.00027.004. https://distill.pub/selforg/2021/adversarial.

[21] Lorenzo Cavuoti, Francesco Sacco, Ettore Randazzo, and Michael Levin. Adversarial takeover of neural cellular automata. In *The 2022 Conference on Artificial Life*. MIT Press, 2022. doi: 10.1162/isal_a_00521. URL `https://doi.org/10.1162/isal_a_00521`.

[22] Caitlin Grasso and Josh Bongard. Empowered neural cellular automata. In *Proceedings of the Genetic and Evolutionary Computation Conference Companion*. ACM, jul 2022. doi: 10.1145/3520304.3529067. URL `https://doi.org/10.1145%2F3520304.3529067`.

[23] Elias Najarro, Shyam Sudhakaran, Claire Glanois, and Sebastian Risi. Hypernca: Growing developmental networks with neural cellular automata, 2022.

[24] Shyam Sudhakaran, Elias Najarro, and Sebastian Risi. Goal-guided neural cellular automata: Learning to control self-organising systems, 2022.

[25] Alex D. Richardson, Tibor Antal, Richard A. Blythe, and Linus J. Schumacher. Learning spatio-temporal patterns with neural cellular automata. *PLOS Computational Biology*, 20 (4):e1011589, April 2024. ISSN 1553-7358. doi: 10.1371/journal.pcbi.1011589. URL `http://dx.doi.org/10.1371/journal.pcbi.1011589`.

[26] Ehsan Pajouheshgar, Yitao Xu, Alexander Mordvintsev, Eyvind Niklasson, Tong Zhang, and Sabine Süsstrunk. Mesh neural cellular automata, 2024. URL `https://arxiv.org/abs/2311.02820`.

[27] James Stovold. Neural cellular automata can respond to signals, 2023.

[28] Ritu Pande and Daniele Grattarola, editors. *Hierarchical Neural Cellular Automata*, volume ALIFE 2023: Ghost in the Machine: Proceedings of the 2023 Artificial Life Conference, 07 2023. doi: 10.1162/isal_a_00601. URL `https://doi.org/10.1162/isal_a_00601`.

[29] Arend Hintze, Mustafa Al-Hammadi, and Eric Libby, editors. *Gene-Regulated Neural Cellular Automata*, volume ALIFE 2024: Proceedings of the 2024 Artificial Life Conference of *Artificial Life Conference Proceedings*, 07 2024. doi: 10.1162/isal_a_00761. URL `https://doi.org/10.1162/isal_a_00761`.

[30] K Sander. Pattern specification in the insect embryo. *Ciba Foundation symposium*, 0(29): 241—263, 1975. ISSN 0300-5208. doi: 10.1002/9780470720110.ch12. URL `https://doi.org/10.1002/9780470720110.ch12`.

[31] Haldo Spontón and Juan Cardelino. A review of classic edge detectors. *Image Processing On Line*, 5:90–123, June 2015. doi: 10.5201/ipol.2015.35. URL `https://doi.org/10.5201/ipol.2015.35`.
